# Aphyllophoroid fungi in insular woodlands of eastern Ukraine

**DOI:** 10.3897/BDJ.5.e22426

**Published:** 2017-12-22

**Authors:** Alexander Ordynets, Anton Savchenko, Alexander Akulov, Eugene Yurchenko, Vera F. Malysheva, Urmas Kõljalg, Josef Vlasák, Karl-Henrik Larsson, Ewald Langer

**Affiliations:** 1 Department of Ecology, University of Kassel, Kassel, Germany; 2 Institute of Ecology and Earth Sciences, University of Tartu, Tartu, Estonia; 3 V.N. Karazin Kharkiv National University, Kharkiv, Ukraine; 4 Department of Biotechnology, Paleski State University, Pinsk, Belarus; 5 Komarov Botanical Institute of the Russian Academy of Sciences, Saint Petersburg, Russia; 6 Biology Centre of the Czech Academy of Sciences, České Budějovice, Czech Republic; 7 Department of Research and Collections, University of Oslo, Natural History Museum, Oslo, Norway

**Keywords:** Basidiomycota, Agaricomycetes, diversity, checklist, wood-inhabiting fungi, ectomycorrhizal fungi, corticioid fungi, polypore fungi, substrate, dead wood, Kharkiv, Donetsk, Luhansk, Nature Reserve, National Park

## Abstract

**Background:**

Fungi play crucial roles in ecosystems and are among the species-richest organism groups on Earth. However, knowledge on their occurrence lags behind the data for animals and plants. Recent analyses of fungal occurrence data from Western, Central and Northern Europe provided important insights into response of fungi to global warming. The consequences of the global changes for biodiversity on a larger geographical scale are not yet understood. Landscapes of Eastern Europe and particularly of eastern Ukraine, with their specific geological history, vegetation and climate, can add substantially new information about fungal diversity in Europe.

**New information:**

We describe the dataset and provide a checklist of aphyllophoroid fungi (non-gilled macroscopic *Basidiomycota*) from eastern Ukraine sampled in 16 areas between 2007 and 2011. The dataset was managed on the PlutoF biodiversity workbench (http://dx.doi.org/10.15156/BIO/587471) and can also be accessed via Global Biodiversity Information Facility (GBIF, parts of datasets https://doi.org/10.15468/kuspj6 and https://doi.org/10.15468/h7qtfd). This dataset includes 3418 occurences, namely 2727 specimens and 691 observations of fructifications belonging to 349 species of fungi. With these data, the digitised CWU herbarium (V. N. Karazin Kharkiv National University, Ukraine) doubled in size A most detailed description of the substrate's properties and habitat for each record is provided. The specimen records are supplemented by 26 nuclear ribosomal DNA ITS sequences and six 28S sequences. Additionally, 287 photographs depicting diagnostic macro- and microscopic features of fungal fruitbodies as well as studied habitats are linked to the dataset. Most of the specimens have at least one mention in literature and relevant references are displayed as associated with specimen data. In total, 16 publication references are linked to the dataset. The dataset sheds new light on the fungal diversity of Eastern Europe. It is expected to complement other public sources of fungal occurrence information on continental and global levels in addressing macroecological and biogeographical questions.

## Introduction

Fungi play crucial roles in ecosystems and are among the species-richest organism groups on Earth (Mora et al. 2011, Heilmann-Clausen et al. 2014, Peay et al. 2016). However, their occurrence has been poorly documented so far due to the difficulties of species detection, identification and delimitation. The situation is now rapidly changing due to substantial improvements in the methods used to identify and communicate the taxa of fungi (Kõljalg et al. 2013, Hibbett et al. 2016). On the other hand, possibilities to digitise the taxon occurrences during the few last years have been additionally improved, providing great benefits for all biodiversity researchers including mycologists (Abarenkov et al. 2010, Senderov et al. 2016).

Europe is the continent with the most advanced knowledge of fungal diversity due to a long-standing tradition of mycological research (Dahlberg et al. 2010). In the last decades, numerous national projects documenting fungal diversity have been initiated. Furthermore, national efforts have been consolidated into the international projects. Such cooperation enables researchers to investigate important ecological questions, for example, species- and community-level responses of fungi to global warming (Andrew et al. 2016, Kauserud et al. 2012). Answering macroecological questions may be facilitated by broad spatial coverage of the dataset, as exemplified by "Climate Change Impacts on the Fungal Ecosystem Component" project,ClimFun (Andrew et al. 2017). There is a place to further expand geographic coverage of successful projects such as ClimFun, but this requires filling sampling gaps and digitisation efforts for national datasets, especially from Eastern Europe. In this region, sparse data availability and accessibility generally result from low numbers of both professional mycologists and citizen scientists (Dahlberg et al. 2010).

The environment of eastern Ukraine (Kharkiv, Donetsk and Luhansk regions) offers a special possibility to study fungal diversity associated with woody plants. Severe continental climate substantially limits the distribution of forests on the local scale, resulting in forest patches of limited size. These "forest oases" are separated from each other by the steppe vegetation or human-managed lands (Popovych 1990). Unlike the other parts of Ukraine and Europe, the European beech and Norway spruce are totally absent in the forests, while pedunculate oak and other numerous deciduous trees form a forest canopy. A specific geological history of the region resulted in the development of forest habitats on chalk outcrops or sandy sediments which are unique on a European scale (Fedorova 1980, Onyshchenko et al. 2007, Didukh and Pashkevich 2003).

Fungal diversity of eastern Ukraine remains insufficiently known. The first scanty documentation of fungal occurrences dates back to the beginning of 19th century (Akulov et al. 2003). The first inventory, specifically focused on the region, was completed four decades ago (Wasser and Soldatova 1977). Less than a decade ago, we reported the results of aphyllophoroid fungi species inventories in several protected areas of eastern Ukraine (Ordynets and Akulov 2011, Ordynets and Akulov 2012, Ordynets et al. 2012, Ordynets et al. 2013). However, these were mostly species lists with sparse metadata, spread over several "floristic papers". This valuable information on fungal occurrences hardly meets the criteria of accessibility, reusability and sharing claimed for biodiversity data nowadays (Senderov and Penev 2016, Costello et al. 2013). Moreover, during the last four years, specimens collected by us were involved in a series of taxonomic studies and were re-identified. All the past and future identifications of the specimens represent valuable information which ideally should be easily updated and traced. Finally, the research area is affected by military activity for more than three years (Vasyliuk et al. 2015) and its preservation and accessibility for research in the forthcoming decades is questionable. Therefore, a proper preservation of the currently available data is required. In this data paper, we describe the effort of digitising and sharing the dataset of aphyllophoroid fungi in selected areas of eastern Ukraine according to the current standards of publishing biodiversity information.

## General description

### Additional information

The project focuses on the diversity of aphyllophoroid fungi. These fungi form neither an evolutionary nor an ecological group but are often targeted as a research object because of both strong taxonomic tradition and sampling convenience. During most of the 20th century, fungi with macroscopic fruitbodies were taxonomically classified according to their fruitbody morphologies. Several generations of mycologists were trained using the morphological classification of fungi. Though these morphological groups barely represent monophyletic taxa and are usually the result of convergent evolution (Hibbett 2007), the present-day identification keys for macrofungi for practical reasons are still compiled based on the principal fruitbody type (e.g. Bernicchia and Gorjón 2010, Krieglsteiner and Kaiser 2000, Ryvarden and Melo 2014).

Aphyllophoroid fungi represent those basidial macrofungi which do not develop gills or closed reproductive structures but have smooth, toothed, irregularly folded to poroid hymenophore and one-celled basidia. They were previously treated as a single taxonomic order but are now found among ca. 20 orders mostly of the class Agaricomycetes (Kirk et al. 2008, Hibbett et al. 2014). Aphyllophoroid fungi are among the best-known groups of fungi globally and especially in Europe (Bernicchia and Gorjón 2010, Ryvarden and Melo 2014). They comprise a highly diverse group both in terms of species richness and functional differentiation. They are the most important agents of wood decay globally (Stokland et al. 2012), but also include mycorrhizal species, plant pathogens and litter saprotrophs (Tedersoo and Smith 2013). In general, aphyllophoroid fungi are strongly dependent on woody plants in terms of nutrition and habitat.

## Project description

### Study area description

Within the project, we inventoried aphyllophoroid fungi on 16 sampling areas located in eastern Ukraine and covering parts of three administrative regions, namely Kharkiv, Donetsk and Luhansk. All sampling areas are located in the middle basin of Siverskyi Donets River, Black Sea basin and their geomorphology developed due to the erosive activity of the river on the massive Upper Cretaceous sediments. We focused on the inventory of the well-preserved areas. We carried out the inventory in two Nature Reserves, i.e. the most strictly protected areas according to the Ukrainian conservational legislation (3 sampling areas), one National Nature Park (8 sampling areas) and one Regional Landscape Park (one sampling area). Four areas had no protected status but were located close to the protected ones. The list of areas with their definition, short characteristics including protection status, links to the areas as PlutoF objects and visiting/sampling statistics are provided in the Table 1. Beside the text description, each area was captured in photographs viewable on the respective area page in PlutoF (see web links in Table 1)

The sampling areas lie in the East European Plain. The elevation varies between 35 and 220 m a.s.l. The Upper Cretaceous sediments (of a chalk and marl) form a series of hills along the right bank of the Siverskyi Donets River, often with outcroppings. Quaternary sediments were formed mostly on the left bank of the river as alluvial and massive sandy accumulations (Bondarchuk 1959). The soils vary according to the bedrock characteristics. On the products of eolation, the ordinary chernozems with medium humus contents were formed. Slopes of the river valley and ravines are covered with the leached sod-calcareous soils. The river floodplain is dominated by meadow chernozems. Alluvial sand accumulations of the second river terrace bear sod-podzolic soils (Didukh and Pashkevich 2003).

According to the Köppen climate classification, the region falls into the area of a cold forest climate with severe winters and dry, long and hot summers (Jylhä et al. 2010). Compared to the rest of Ukraine, here the climate has the most pronounced continental characteristics. The mean annual temperature is 7.5°С, with recorded minimum of -40°С (January) and maximum of 39°С (July). The average annual precipitation is 400‒540 mm, while the evaporation is 580‒650 mm. Up to 60 days per year with very strong hot dry winds are possible (Popov et al. 1968).

According to the classification of terrestrial ecoregions of the world, the study area belongs to the biome of temperate grasslands, savannahs and shrublands (Olson et al. 2001). It is also known as a steppe region (EEA 2015). In these conditions, the natural woody vegetation mostly represents forest patches of limited size. The few striking exceptions exist due to their proximity to the large Siverskyi Donets River, as well as hilly landscape. Two broadleaf forests (called Teplynska dacha and Maiatska dacha) are the largest forest massifs on the watershed in the Ukrainian steppe zone (Onyshchenko et al. 2007).

The pedunculate oak *Quercus robur* L. is one of the most important tree species in the area. In the forests on the watershed, this oak species is accompanied by *Fraxinus excelsior* L., *Tilia cordata* Mill., *Acer platanoides* L., *A. campestre* L., *A. tataricum* L. and *Corylus avellana* L. In the Siverskyi Donets floodplain, the oak forest grows mosaically with the forests composed of *Alnus glutinosa* (L.) Gaertn., various poplars (*Populus nigra* L., *P. alba* L. and *P. tremula* L.), willows (*Salix alba* L., *Salix fragilis* L.) and *Acer negundo* L. (Popovych 1990).

The massive sandy terrace of the Siverskyi Donets River is basically a habitat for the psammophytic grasses and herbs. However, there are numerous depressions which provide a suitable microclimate for the development of the wetland local forests (groves). The usual trees in these habitats are: *Betula pendula* Roth, *B. pubescens* Ehrh., *Populus tremula*, *P. nigra* and *P. alba*, *Alnus glutinosa* and *Crataegus* spp. The willow shrubby communities of *Salix acutifolia* Willd. and *Salix rosmarinifolia* L. are common in some localities (Popovych 1990). As a result of land-use in the 19th and 20th centuries, large areas of the sandy terrace are currently covered not by primary vegetation of psammophytic grasses and herbs, but by plantations of *Pinus sylvestris* L. However, some of these stands are about 200 years old and resemble natural forests. The true natural pine forests in the region are currently confined to the chalky outcrops with the sod-calcareous soils. These populations were treated in the past decades as a relict species *Pinus cretacea* Kalen. (Didukh and Pashkevich 2003, Fedorova 1980) currently regarded as a variety of Scots pine, P. sylvestris var. cretacea (Kalen.) Kom. (Gardner 2013).

### Funding

The work of Alexander Ordynets and Alexander Akulov in eastern Ukraine was supported by the V.N. Karazin Kharkiv National University and Ukrainian Ministry of Education and Science Grant Number 0112U007570/16-16-13 “Biodiversity exploration as a base for the nature conservation network development in Ukraine”. Alexander Ordynets and Ewald Langer were supported by the University of Kassel and the programme LOEWE (“Landes-Offensive zur Entwicklung Wissenschaftlich-ökonomischer Exzellenz”) of the Hesse's Ministry of Higher Education, Research and Arts within the framework of the Integrative Fungal Research (IPF) project (http://www.integrative-pilzforschung.de). The work of Alexander Ordynets on the molecular identification of Thelephorales was undertaken due to support from the European Social Fund’s Doctoral Studies and Internationalisation Programme DoRa and hosted by the Chair of Mycology, Institute of Ecology and Earth Sciences, University of Tartu, Estonia.

## Sampling methods

### Sampling description

In 16 areas, we sampled living fungal fruitbodiesaccording to the standards for macrofungi collecting (Lodge et al. 2004). We were interested in recording the majority of species from the local species pool. Therefore we sampled not within fixed areas, but covered larger areas by sampling along the forest paths. We were passing up to ten kilometres of forest paths per day. We spent more time at the points which visually harboured higher amounts and diversity of deadwood. Visiting and sampling statistics for each area are provided in Table 1.

If the species could be readily identified in the field, the occurrence was recorded without taking a specimen, i.e. as observation, except for the very first finding of the species during the field work. All fruiting bodies from a single woody substrate or growing in a single cluster on debris or litter were treated as a single individual. For the records on woody substrata (which prevail in the dataset), principal tree fraction, diameter class, decay stage, spatial location and presence/absence of the direct contact with soil were recorded. For all the records, plant species of the substrate as well as characteristics of forest habitat and mesorelief were described (Table 2). All collected data were uploaded and managed on the PlutoF platform (Abarenkov et al. 2010).

### Quality control

One of the challenges in inventorying fungal diversity based on fruiting bodies is the temporal irregularity of fruiting. To reduce the bias associated with this phenomenon, within five years (2007‒2011), we carried out 15 expeditions, usually making one expedition per season (spring, summer and autumn). The earliest and latest sampling dates were March 9 and November 22 respectively. Each expedition lasted as a minimum three and as a maximum seven days. Within a single expedition, we visited up to four sampling areas (see Table 1). Some areas could be visited only once. This may be acceptable in the case of aphyllorphoid fungi which, as opposed to other fruiting fungi, in general create more lasting fruitbodies.

Along with the specimens which were more or less easily identified, there were also collections whose identifications were verified by another expert or collections which we could identify to the genus level only. Wolfgang Dämon, Ivan Zmitrovich, Anton Shyriaev, Heikki Kotiranta, Masoomeh Ghobad-Nejhad, Philomena Bodensteiner, Sergey Volobuev, Viacheslav Spirin and Erast Parmasto kindly helped us in such issues. The names of the experts who verified or improved our identifications are provided in the pane "Identifications", fields "Identifiers" and/or "Remarks" on the respective specimen page as viewed in PlutoF. Additionally, each PlutoF observation linked to our dataset was verified by the platform developer as seen in the pane "Discussion" on the single observation page.

### Step description

Noticeable specimens were photographed directly in the field or after drying in the laboratory. The micromorphology study of the dried specimens was performed under 1000× magnification using light microscopes Zeiss Primo Star (Carl Zeiss Jena GmbH, Jena, Germany) and Nicon Eclipse 90i (Nikon Corp., Tokyo, Japan). The specimens were examined in 5% aqueous potassium hydroxide solution, Melzer’s reagent and 1% Congo red solution in concentrated ammonia (Ryvarden and Melo 2014). The main identification keys used were Bernicchia and Gorjón (2010), Kõljalg (1996), Larsson (1992), Ryvarden and Melo (2014).

## Geographic coverage

### Description

The extent of all sampling areas covers about 180 km in the longitudinal and 60 km in latitudinal direction. The total area of study is 380 km^2^.

### Coordinates

48.7175° and 49.29° Latitude; 36.91884° and 39.39385° Longitude.

## Taxonomic coverage

### Description

The dataset contains 3418 species occurrences. It has 2727 specimens and 691 observation records, containing 349 species of fungi from the phylum Basidiomycota. Additional 16 items have no specific epithet but only genus-level (in one case order-level) identification (Table 3).

Most of the species belong to subphylum Agaricomycotina, class Agaricomycetes and represent 14 orders (Agaricales, Atheliales, Amylocorticiales, Auriculariales, Cantharellales, Boletales, Gloeophyllales, Corticiales, Hymenochaetales, Gomphales, Polyporales, Russulales, Thelephorales and Trechisporales). One genus belongs to a separate lineage of presumably order level (*Xenasmatella* *spp.*) and five species have an unclear position within Agaricomycetes (three *Dendrothele* *spp.*, *Granulobasidium vellereum* and *Trechinothus smardae*). Three species in the dataset represent the subphylum Pucciniomycotina: *Helicogloea farinacea* and *H. lagerheimii* (Atractiellales, Atractiellomycetes) and *Colacogloea peniophorae* (Microbotryomycetes).

The dataset includes several species described or raised to the species level status in the the last two decades: *Antrodiella ichnusana*, *Lyomyces erastii*, *L. incrustatus, Phlebia tuberculata* and *Xylodon tuberculatus*. We could use these names for identification purposes by 2012, i.e. by the end of specimen identification phase of the project. However, to some specimens, new important identifications were added in the course of taxonomic revisions published after 2011 (Table 4). Further important recent nomenclatural innovations reflected in the dataset are treating *Tomentella crinalis* and *T. fibrosa* in the separate genus *Odontia*, following Tedersoo et al. (2014).

From 19 specimens representing 16 species, we generated 19 nuclear ribosomal DNA ITS sequences and four 28S sequences. Further seven ITS and two 28S sequences were produced in collaborative studies focusing on a particular taxonomic problem (see Table 4). The sequences can be found in the public repositories UNITE (Kõljalg et al. 2013) and GenBank (Benson et al. 2013) and linked to the respective CWU vouchers in PlutoF. The sequence UDB033929*1 from specimen CWU4336*1 is the first barcode sequence for the species *Phellinus rhamni.* Our photographs of *Dichomitus squalens* (CWU6509) and *Lenzites warnieri* (CWU6505) linked to the dataset illustrate the respective species in the latest key to European polypores (Ryvarden and Melo 2014).

## Traits coverage

The assignments to the 1) lifestyle and 2) fruiting-body principal configuration (morph) type were provided for each species. Lifestyle is a predefined field in PlutoF, from which we used the categories saprotroph, symbiotroph and parasite.

The principal fruitbody configuration of macrofungi is an increasingly addressed species trait in the ecological and evolutionary studies (Abrego et al. 2016, Hibbett 2007). We classified the species of our datasetinto the following groups: those where fruitbodies have smooth spore-producing surface (corticiod), species with cup-shaped fruitbodies (cyphelloid), species with toothed hymenophore (hydnoid), species developing pores (poroid), species having gelified fruiting bodies (heterobasidial) and species having coralloid, club-like or funnel-like fruiting bodies which grow negatively geotropic (clavarioid). As the trait module for fungi in PlutoF is still under development (July 2017), we specified the fruiting body morph in the field "Identifications.Remarks" for each specimen and observation.

## Temporal coverage

**Data range:** 2007-3-09 – 2011-5-07.

## Collection data

### Collection name

All the specimens are stored in the V.N. Karazin National University Herbarium, Kharkiv, Ukraine (CWU). Many specimens belonging to the order Thelephorales are recorded in PlutoF having their main deposition place as: University of Tartu; Natural History Museum and Botanic Garden; Botanical and Mycological Museum; Department of Mycology, TU(M). The duplicates of CWU specimens studied by colleagues were placed in the herbaria of their institutions: M.G. Kholodny Institute of Botany, National Academy of Sciences of Ukraine (KW), V.F. Kuprevich Institute of Experimental Botany, Belarus Academy of Sciences (MSK), V.L. Komarov Botanical Institute (LE) and Institute of Plant and Animal Ecology (SVER) of the Russian Academy of Sciences, University of Gothenburg (GB) and personal collections of Josef Vlasák, Wolfgang Dämon, Heikki Kotiranta and Masoomeh Ghobad-Nejhad (will join the Iranian Cryptogamic Herbarium, ICH).

### Collection identifier

CWU, TU(M), KW, MSK, LE, SVER, GB, ICH.

### Specimen preservation method

Fresh specimens were dried with an electric fan dryer on the day of collection and placed in grip seal plastic bags. Shortly after drying, the specimens were placed into a deep freezer (‒20°C) for a week, to prevent their destruction by insects. Specimens are preserved in cardboard herbarium boxes.

### Curatorial unit

CWU fungal specimens are curated by the Department of Mycology and Plant Resistance of V.N. Karazin Kharkiv National University, Ukraine.

## Usage rights

### Use license

Open Data Commons Attribution License

### IP rights notes

The dataset is hosted by PlutoF and accessible from the latter under Attribution-NonCommercial-ShareAlike 4.0 International License (CC BY-NC-SA 4.0). The source records compiled into the dataset are available in the dedicated PlutoF project (https://plutof.ut.ee/#/study/view/38925). Project specimens and sequences are open for alternative taxon identifications. The occurrence records may also be viewed in the GBIF occurrence dataset of CWU herbarium (Savchenko 2017, https://doi.org/10.15468/kuspj6) and the GBIF dataset of PlutoF platform observations (PlutoF 2017, https://doi.org/10.15468/h7qtfd). All data resources are also provided in Suppl. material 1.

## Data resources

### Data package title

Aphyllophoroid fungi in insular woodlands of eastern Ukraine.

### Resource link

http://dx.doi.org/10.15156/BIO/587471

### Alternative identifiers

https://plutof.ut.ee/#/study/view/38925

### Number of data sets

3

### Data set 1.

#### Data set name

Specimens

#### Data format

Darwin Core Archive

#### Number of columns

1

#### Character set

UTF-8

#### Download URL

https://data.datacite.org/application/zip/10.15156/BIO/587471

#### Data format version

1.0

#### Description

Specimens of aphyllophoroid fungi (non-gilled macroscopic Basidiomycota) from eastern Ukraine collected between 2007 and 2011.

**Data set 1.** 

Column label	Column description
http://rs.tdwg.org/dwc/terms/	See terms in the link

### Data set 2.

#### Data set name

Observations

#### Data format

Darwin Core Archive

#### Number of columns

1

#### Character set

UTF-8

#### Download URL

https://data.datacite.org/text/zip-1072799/10.15156/BIO/587471

#### Data format version

1.0

#### Description

Observations of aphyllophoroid fungi (non-gilled macroscopic Basidiomycota) from eastern Ukraine made between 2007 and 2011.

**Data set 2.** 

Column label	Column description
http://rs.tdwg.org/dwc/terms/	See terms in the link

### Data set 3.

#### Data set name

Areas shape files

#### Data format

ESRI Shapefile

#### Number of columns

2

#### Character set

UTF-8

#### Download URL

http://data.datacite.org/text/x-rar/10.15156/BIO/587471

#### Description

Shapefile of all samping areas used in the study

**Data set 3.** 

Column label	Column description
id	Short identifier without whitespaces (text string)
name	Full names of areas (text string)

## Additional information

### Results communication

The results of species inventories in eastern Ukraine were communicated and discussed at several conferences and meetings attended by Alexander Ordynets and Alexander Akulov:

- Conferences for young scientists organised by M.G. Kholodny Institute of Botany, National Academy of Sciences of Ukraine (2008 in Kamianets-Podilskyi, 2009 in Kremianets, 2010 and 2011 in Kiev, Ukraine);
- Conferences for young scientists organised by V.N. Karazin National University in Kharkiv, Ukraine (2008-2011);
- Presentations on Ukrainian fungi at the Microbial Evolution Research Group, University of Oslo (2012-10-16) and Botanical Museum, University of Oslo (2014-03-27);
- Field course on identification of corticioid Basidiomycetes (2012-09-10 ‒ 2012-09-15) and winter seminar (2013-02-25), organised by the Chair of Mycology, Institute of Ecology and EarthSciences, University of Tartu, Estonia;
- Presentation on Ukrainian fungi diversity at the Department of Mycology, University of Marburg, Germany (2013-06-17).

### Outlook

While uploading and processing our data in the PlutoF system, we found that some species were missing in the PlutoF classification. Therefore we added manually to the PlutoF taxonomy one genus (*Heteroradulum*) and the six following species: *Basidiodendron deminutum*, *B. rimulentum*, *Heteroradulum kmetii*, *Hyphodontia incrustata*, *Maireina maxima* and *Trechinothus smardae*. Two more species, *Sistotremella hauerslevii* and *Henningsomyces stipitatus*, were added to PlutoF classification by means of import from the GBIF Backbone Taxonomy of 2016. One plant species, *Prunus stepposa*, was also manually added to the Plantae kingdom of PlutoF taxonomy. These taxon additions will ease the data upload for subsequent PlutoF users.

Our data contribute to more than a doubling in size of the digitised CWU herbarium, the first and the largest digital collection outside Estonia hosted by PlutoF (Savchenko 2017). Our dataset sheds further light on the fungal diversity of Eastern Europe and it is anticipated that it will complement other data sources on European fungi in addressing macroecological and biogeographical questions. It is also hoped that the example of this data paper will promote further effective enrichment of PlutoF platform with fungal occurrence data.

## Supplementary Material

Supplementary material 1 Data resources: Aphyllophoroid fungi in insular woodlands of eastern Ukraine Data type: occurrences, links to multimedia
Brief description: Seperate Darwin Core Archives for specimens and observations, plus shape files of sampling areas.
File: oo_173713.zip Alexander Ordynets, Anton Savchenko, Alexander Akulov, Eugene Yurchenko, Vera Malysheva, Urmas Kõljalg, Josef Vlasák, Karl-Henrik Larsson, Ewald Langer

## Figures and Tables

**Table 1.** Definitions of 16 sampling areas in eastern Ukraine where aphyllophoroid fungi were inventoried, with number of visits and taxa occurrences recorded.

**Sampling area name**	**Locality text**	**Region**	**Commune**	URL to the area in PlutoF*^1^	**Number of visits to area**	**Number of specimens** **collected**	**Number of observations**
Ukraine, Iziumska Luka floodplain	Iziumska Luka Regional Landscape Park, floodplain of the Siverskyi Donets River	Kharkiv	Balaklea	https://plutof.ut.ee/#/area/view/1782438	2	588	25
Ukraine, Iziumska Luka sandy terrace	Large sandy massif to the east from Iziumska Luka Regional Landscape Park	Kharkiv	Balaklea	https://plutof.ut.ee/#/area/view/1782428	2	78	11
Ukraine, Yaremivka	Area between Siverskyi Donets River and railway Izium-Sviatohirsk, between "Bukino" and "Studenok" train stations, including the candidate protected area Yaremivskyi Preserve	Kharkiv	Izium	https://plutof.ut.ee/#/area/view/1782489	2	74	43
Ukraine, Sviatohirsk floodplain	Sviati Hory National Nature Park, floodplain to the south of Sviatohirsk town	Donetsk	Sloviansk	https://plutof.ut.ee/#/area/view/1782490	7	127	39
Ukraine, Sviatohirsk hills	Sviati Hory National Nature Park, high steep hills south of the Syverskyi Donets River	Donetsk	Sloviansk	https://plutof.ut.ee/#/area/view/1782491	4	75	34
Ukraine, Sviatohirsk sandy terrace	Sviati Hory National Nature Park, pinery with inclusion of deciduous forest patches to the north and east of Sviatohirsk town	Donetsk	Lyman	https://plutof.ut.ee/#/area/view/1782495	8	610	206
Ukraine, Teplynske	Sviati Hory National Nature Park, west of Bogorodychne village	Donetsk	Sloviansk	https://plutof.ut.ee/#/area/view/1782493	5	115	149
Ukraine, Maiatske	Sviati Hory National Nature Park, west of Maiaki village	Donetsk	Sloviansk	https://plutof.ut.ee/#/area/view/1782494	2	85	5
Ukraine, Drobyshivske	Sviati Hory National Nature Park, forest south-west of Drobyshevo village	Donetsk	Lyman	https://plutof.ut.ee/#/area/view/1782496	3	52	110
Ukraine, Lyman sandy terrace	Sviati Hory National Nature Park, sandy terrace south of Lyman town (before 2016 named Krasnyi Lyman)	Donetsk	Lyman	https://plutof.ut.ee/#/area/view/1782498	1	23	0
Ukraine, Lyman floodplain	Sviati Hory National Nature Park, floodplain south of Brusivka and Dibrova villages	Donetsk	Lyman	https://plutof.ut.ee/#/area/view/1782499	1	6	0
Ukraine, Kreidova flora hills	Kreidova Flora, division of the Ukrainian Steppe Nature Reserve, vicinities of Kryva Luka village	Donetsk	Sloviansk	https://plutof.ut.ee/#/area/view/1782500	1	220	0
Ukraine, Kreidova flora floodplain	Floodplain of the Syverskyi Donets River near Kryva Luka village	Donetsk	Sloviansk	https://plutof.ut.ee/#/area/view/1782501	1	101	0
Ukraine, Trokhizbenka sandy terrace	Trokhizbenskyi Step division of the Luhansk Nature Reserve, north of Trokhizbenka and Kriakivka villages	Luhansk	Slovianoserbsk	https://plutof.ut.ee/#/area/view/1782503	1	136	52
Ukraine, Trokhizbenka floodplain	Floodplain of Syverskyi Donets River south of Trokhizbenka and Kriakivka villages	Luhansk	Slovianoserbsk	https://plutof.ut.ee/#/area/view/1782505	1	11	17
Ukraine, Stanychno-Luhanske	Stanychno-Luhanske division of the Luhansk Nature Reserve, east of Stanytsia Luhanska town	Luhansk	Stanytsia Luhanska	https://plutof.ut.ee/#/area/view/1782502	1	426	0

**Table 2.** Substrate and habitat characteristics of fungal occurrence data recorded in the project.

Variable attribution	Variable	Number of categories	States of the variable	Reference
Substrate: all records	Plant species	56		
Substrate: records from wood	Principal tree fraction	3	branch; stump; trunk	Safonov 2003
	Diameter class	4	1 (<3 cm); 2 (3‒10 cm); 3 (10‒20 cm); 4 (>20 cm), according to measurement at the base of woody unit	Safonov 2003
	Wood decay stage	5	1‒5 according to the depth of knife penetration into the wood, bark cover, preservation of initial circumference	Heilmann-Clausen and Christensen 2003, Renvall 1995
	Spatial location of substrate	3	attached (including suspended); fallen; standing (including snags and stumps)	This project
	Direct contact with soil	2	in contact with soil; no contact with soil	This project
Habitat	Forest spread type	5	continuous; focal in steppe; focal surrounded by artificial pine forest; sparce; windbreaks	This project
	Forest type, according to dominating tree species	6	alder, birch, oak, pinery, poplar, willow	This project
	Mesorelief type	6	floodplain, ravine, sands depression, sands rising, steep river bank, watershed	Belgard 1971

**Table 3.** Species checklist and frequencies of occurrence of aphyllophoroid fungi (phylum Basidiomycota) from insular woodlands of eastern Ukraine. Specific epithet is available for 349 taxa, while it could not be found for 16 items (only genus-level or order-level identification was possible)

**Taxon name**	**Count speci-mens**	**Count obser-vations**	**Class**	**Order**	**Family**	**Genus**
*Abortiporus biennis*	1		Agaricomycetes	Polyporales	Podoscyphaceae	Abortiporus
*Aleurodiscus dextrinoideocerussatus*	2		Agaricomycetes	Russulales	Stereaceae	Aleurodiscus
*Amphinema byssoides*	6		Agaricomycetes	Atheliales	Atheliaceae	Amphinema
*Amyloxenasma allantosporum*	1		Agaricomycetes	Amylocorticiales	Amylocorticiaceae	Amyloxenasma
*Antrodia albida*	2		Agaricomycetes	Polyporales	Fomitopsidaceae	Antrodia
*Antrodia gossypium*	9		Agaricomycetes	Polyporales	Fomitopsidaceae	Antrodia
*Antrodia hyalina*	13		Agaricomycetes	Polyporales	Fomitopsidaceae	Antrodia
*Antrodia malicola*	24	4	Agaricomycetes	Polyporales	Fomitopsidaceae	Antrodia
*Antrodia ramentacea*	4		Agaricomycetes	Polyporales	Fomitopsidaceae	Antrodia
*Antrodia sinuosa*	1		Agaricomycetes	Polyporales	Fomitopsidaceae	Antrodia
*Antrodia* sp.	1		Agaricomycetes	Polyporales	Fomitopsidaceae	Antrodia
*Antrodia xantha*	2		Agaricomycetes	Polyporales	Fomitopsidaceae	Antrodia
*Antrodiella faginea*	2		Agaricomycetes	Polyporales	Steccherinaceae	Antrodiella
*Antrodiella ichnusana*	1		Agaricomycetes	Polyporales	Steccherinaceae	Antrodiella
*Antrodiella romellii*	1		Agaricomycetes	Polyporales	Steccherinaceae	Antrodiella
*Aphanobasidium pseudotsugae*	2		Agaricomycetes	Agaricales	Pterulaceae	Aphanobasidium
*Aporpium canescens*	10		Agaricomycetes	Auriculariales	Auriculariales fam incertae sedis	Aporpium
*Artomyces pyxidatus*	5	5	Agaricomycetes	Russulales	Auriscalpiaceae	Artomyces
*Athelia acrospora*	2		Agaricomycetes	Atheliales	Atheliaceae	Athelia
*Athelia arachnoidea*	33	3	Agaricomycetes	Atheliales	Atheliaceae	Athelia
*Athelia bombacina*	2		Agaricomycetes	Atheliales	Atheliaceae	Athelia
*Athelia decipiens*	14		Agaricomycetes	Atheliales	Atheliaceae	Athelia
*Athelia epiphylla*	26		Agaricomycetes	Atheliales	Atheliaceae	Athelia
*Athelia fibulata*	3		Agaricomycetes	Atheliales	Atheliaceae	Athelia
*Athelia salicum*	2		Agaricomycetes	Atheliales	Atheliaceae	Athelia
*Athelia* sp.	11		Agaricomycetes	Atheliales	Atheliaceae	Athelia
*Aurantiporus fissilis*	2	1	Agaricomycetes	Polyporales	Meruliaceae	Aurantiporus
*Auriscalpium vulgare*	3	7	Agaricomycetes	Russulales	Auriscalpiaceae	Auriscalpium
*Basidiodendron caesiocinereum*	1		Agaricomycetes	Auriculariales	Exidiaceae	Basidiodendron
*Basidiodendron deminutum*	2		Agaricomycetes	Auriculariales	Exidiaceae	Basidiodendron
*Basidiodendron eyrei*	8		Agaricomycetes	Auriculariales	Exidiaceae	Basidiodendron
*Basidioradulum tuberculatum*	1		Agaricomycetes	Hymenochaetales	Schizoporaceae	Basidioradulum
*Bjerkandera adusta*	19	14	Agaricomycetes	Polyporales	Meruliaceae	Bjerkandera
*Bjerkandera fumosa*	2	1	Agaricomycetes	Polyporales	Meruliaceae	Bjerkandera
*Boidinia furfuracea*	1		Agaricomycetes	Russulales	Russulales fam incertae sedis	Boidinia
*Botryobasidium arachnoideum*	1		Agaricomycetes	Cantharellales	Botryobasidiaceae	Botryobasidium
*Botryobasidium aureum*	2		Agaricomycetes	Cantharellales	Botryobasidiaceae	Botryobasidium
*Botryobasidium candicans*	22		Agaricomycetes	Cantharellales	Botryobasidiaceae	Botryobasidium
*Botryobasidium conspersum*	34		Agaricomycetes	Cantharellales	Botryobasidiaceae	Botryobasidium
*Botryobasidium curtisii*	3		Agaricomycetes	Cantharellales	Botryobasidiaceae	Botryobasidium
*Botryobasidium laeve*	12		Agaricomycetes	Cantharellales	Botryobasidiaceae	Botryobasidium
*Botryobasidium pruinatum*	10		Agaricomycetes	Cantharellales	Botryobasidiaceae	Botryobasidium
*Botryobasidium robustius*	8		Agaricomycetes	Cantharellales	Botryobasidiaceae	Botryobasidium
*Botryobasidium simile*	1		Agaricomycetes	Cantharellales	Botryobasidiaceae	Botryobasidium
*Botryobasidium sphaericosporum*	8		Agaricomycetes	Cantharellales	Botryobasidiaceae	Botryobasidium
*Botryobasidium subcoronatum*	46		Agaricomycetes	Cantharellales	Botryobasidiaceae	Botryobasidium
*Botryohypochnus isabellinus*	7		Agaricomycetes	Cantharellales	Botryobasidiaceae	Botryohypochnus
*Bourdotia galzinii*	1		Agaricomycetes	Auriculariales	Auriculariales incertae sedis	Bourdotia
*Brevicellicium olivascens*	1		Agaricomycetes	Trechisporales	Hydnodontaceae	Brevicellicium
*Bulbillomyces farinosus*	8		Agaricomycetes	Polyporales	Podoscyphaceae	Bulbillomyces
*Byssomerulius corium*	23	10	Agaricomycetes	Polyporales	Meruliaceae	Byssomerulius
*Ceraceomyces serpens*	12		Agaricomycetes	Polyporales	Meruliaceae	Ceraceomyces
*Ceraceomyces tessulatus*	3		Agaricomycetes	Polyporales	Meruliaceae	Ceraceomyces
*Ceratobasidium cornigerum*	10		Agaricomycetes	Cantharellales	Ceratobasidiaceae	Ceratobasidium
*Ceriporia purpurea*	19	1	Agaricomycetes	Polyporales	Meruliaceae	Ceriporia
*Ceriporia reticulata*	1		Agaricomycetes	Polyporales	Meruliaceae	Ceriporia
*Ceriporia viridans*	4		Agaricomycetes	Polyporales	Meruliaceae	Ceriporia
*Ceriporiopsis mucida*	1		Agaricomycetes	Polyporales	Steccherinaceae	Ceriporiopsis
*Ceriporiopsis resinascens*	3		Agaricomycetes	Polyporales	Steccherinaceae	Ceriporiopsis
*Cerrena unicolor*	8	3	Agaricomycetes	Polyporales	Cerrenaceae	Cerrena
*Chondrostereum purpureum*	10	13	Agaricomycetes	Agaricales	Agaricales fam incertae sedis	Chondrostereum
*Colacogloea peniophorae*	3		Microbotryomycetes	Microbotryomycetes ord incertae sedis	Microbotryomycetes fam incertae sedis	Colacogloea
*Coltricia perennis*	1	1	Agaricomycetes	Hymenochaetales	Hymenochaetaceae	Coltricia
*Coniophora arida*	36	3	Agaricomycetes	Boletales	Coniophoraceae	Coniophora
*Coniophora fusispora*	4		Agaricomycetes	Boletales	Coniophoraceae	Coniophora
*Coniophora olivacea*	13		Agaricomycetes	Boletales	Coniophoraceae	Coniophora
*Coniophora puteana*	31	3	Agaricomycetes	Boletales	Coniophoraceae	Coniophora
*Coniophora* sp.	1		Agaricomycetes	Boletales	Coniophoraceae	Coniophora
*Coriolopsis gallica*	3		Agaricomycetes	Polyporales	Polyporaceae	Coriolopsis
*Corticium roseum*	10		Agaricomycetes	Corticiales	Corticiaceae	Corticium
*Cristinia helvetica*	1		Agaricomycetes	Agaricales	Stephanosporaceae	Cristinia
*Cristinia rhenana*	24		Agaricomycetes	Agaricales	Stephanosporaceae	Cristinia
*Cylindrobasidium evolvens*	7		Agaricomycetes	Agaricales	Physalacriaceae	Cylindrobasidium
*Dacryobolus sudans*	3		Agaricomycetes	Polyporales	Fomitopsidaceae	Dacryobolus
*Daedalea quercina*	4	8	Agaricomycetes	Polyporales	Fomitopsidaceae	Daedalea
*Daedaleopsis confragosa*	3	4	Agaricomycetes	Polyporales	Polyporaceae	Daedaleopsis
*Datronia mollis*	1		Agaricomycetes	Polyporales	Polyporaceae	Datronia
*Dendrothele acerina*	10	8	Agaricomycetes	Agaricomycetes ord incertae sedis	Agaricomycetes fam incertae sedis	Dendrothele
*Dendrothele alliacea*	15	4	Agaricomycetes	Agaricomycetes ord incertae sedis	Agaricomycetes fam incertae sedis	Dendrothele
*Dendrothele minutissima*	1		Agaricomycetes	Agaricomycetes ord incertae sedis	Agaricomycetes fam incertae sedis	Dendrothele
*Dichomitus campestris*	7	4	Agaricomycetes	Polyporales	Polyporaceae	Dichomitus
*Dichomitus squalens*	16	1	Agaricomycetes	Polyporales	Polyporaceae	Dichomitus
*Dichostereum effuscatum*	1		Agaricomycetes	Russulales	Lachnocladiaceae	Dichostereum
*Diplomitoporus flavescens*	6		Agaricomycetes	Polyporales	Meripilaceae	Diplomitoporus
*Erythricium hypnophilum*	2		Agaricomycetes	Corticiales	Corticiaceae	Erythricium
*Exidiopsis galzinii*	1		Agaricomycetes	Auriculariales	Exidiaceae	Exidiopsis
*Exidiopsis griseobrunnea*	1		Agaricomycetes	Auriculariales	Exidiaceae	Exidiopsis
*Fibriciellum silvae-ryae*	1		Agaricomycetes	Trechisporales	Hydnodontaceae	Fibriciellum
*Fibricium subceraceum*	3		Agaricomycetes	Polyporales	Steccherinaceae	Fibricium
*Fibrodontia gossypina*	2		Agaricomycetes	Trechisporales	Hydnodontaceae	Fibrodontia
*Fibroporia vaillantii*	1		Agaricomycetes	Polyporales	Fomitopsidaceae	Fibroporia
*Fibulomyces fusoideus*	6		Agaricomycetes	Atheliales	Atheliaceae	Fibulomyces
*Fibulomyces mutabilis*	5		Agaricomycetes	Atheliales	Atheliaceae	Fibulomyces
*Fibulomyces* sp.	2		Agaricomycetes	Atheliales	Atheliaceae	Fibulomyces
*Fistulina hepatica*	3	2	Agaricomycetes	Agaricales	Fistulinaceae	Fistulina
*Fomes fomentarius*	4	81	Agaricomycetes	Polyporales	Polyporaceae	Fomes
*Fomitiporia punctata*	9	7	Agaricomycetes	Hymenochaetales	Hymenochaetaceae	Fomitiporia
*Fomitiporia robusta*	3	14	Agaricomycetes	Hymenochaetales	Hymenochaetaceae	Fomitiporia
*Fuscoporia contigua*	25	2	Agaricomycetes	Hymenochaetales	Hymenochaetaceae	Fuscoporia
*Fuscoporia ferruginosa*	47	3	Agaricomycetes	Hymenochaetales	Hymenochaetaceae	Fuscoporia
*Galzinia incrustans*	3		Agaricomycetes	Corticiales	Corticiaceae	Galzinia
*Ganoderma applanatum*	7	14	Agaricomycetes	Polyporales	Ganodermataceae	Ganoderma
*Ganoderma lucidum*	1		Agaricomycetes	Polyporales	Ganodermataceae	Ganoderma
*Ganoderma resinaceum*	4		Agaricomycetes	Polyporales	Ganodermataceae	Ganoderma
*Gloeocystidiellum porosum*	3		Agaricomycetes	Russulales	Gloeocystidiellaceae	Gloeocystidiellum
*Gloeohypochnicium analogum*	1		Agaricomycetes	Amylocorticiales	Amylocorticiaceae	Gloeohypochnicium
*Gloeophyllum sepiarium*	2		Agaricomycetes	Gloeophyllales	Gloeophyllaceae	Gloeophyllum
*Gloeophyllum trabeum*	3		Agaricomycetes	Gloeophyllales	Gloeophyllaceae	Gloeophyllum
*Gloeoporus dichrous*	15	17	Agaricomycetes	Polyporales	Meruliaceae	Gloeoporus
*Gloeoporus pannocinctus*	12	2	Agaricomycetes	Polyporales	Meruliaceae	Gloeoporus
*Gloeoporus taxicola*	2	1	Agaricomycetes	Polyporales	Meruliaceae	Gloeoporus
*Gloiothele lactescens*	8		Agaricomycetes	Russulales	Lachnocladiaceae	Gloiothele
*Granulobasidium vellereum*	14		Agaricomycetes	Agaricomycetes ord incertae sedis	Agaricomycetes fam incertae sedis	Granulobasidium
*Hapalopilus nidulans*	6	1	Agaricomycetes	Polyporales	Meruliaceae	Hapalopilus
*Helicogloea farinacea*	1		Atractiellomycetes	Atractiellales	Atractiellales fam incertae sedis	Helicogloea
*Helicogloea lagerheimii*	2		Atractiellomycetes	Atractiellales	Atractiellales fam incertae sedis	Helicogloea
*Henningsomyces candidus*	7		Agaricomycetes	Agaricales	Cyphellaceae	Henningsomyces
*Henningsomyces stipitatus*	1		Agaricomycetes	Agaricales	Cyphellaceae	Henningsomyces
*Hericium coralloides*	2		Agaricomycetes	Russulales	Hericiaceae	Hericium
*Heterobasidion annosum*	1		Agaricomycetes	Russulales	Bondarzewiaceae	Heterobasidion
*Heteroradulum kmetii*	7		Agaricomycetes	Auriculariales	Exidiaceae	Heteroradulum
*Hydnocristella himantia*	4		Agaricomycetes	Gomphales	Lentariaceae	Hydnocristella
*Hymenochaete cinnamomea*	23	5	Agaricomycetes	Hymenochaetales	Hymenochaetaceae	Hymenochaete
*Hymenochaete fuliginosa*	9		Agaricomycetes	Hymenochaetales	Hymenochaetaceae	Hymenochaete
*Hymenochaete rubiginosa*	11	32	Agaricomycetes	Hymenochaetales	Hymenochaetaceae	Hymenochaete
*Hyphoderma argillaceum*	1		Agaricomycetes	Polyporales	Hyphodermataceae	Hyphoderma
*Hyphoderma griseoflavescens*	1		Agaricomycetes	Polyporales	Hyphodermataceae	Hyphoderma
*Hyphoderma mutatum*	14		Agaricomycetes	Polyporales	Hyphodermataceae	Hyphoderma
*Hyphoderma setigerum*	44		Agaricomycetes	Polyporales	Hyphodermataceae	Hyphoderma
*Hyphoderma* sp.	1		Agaricomycetes	Polyporales	Hyphodermataceae	Hyphoderma
*Hyphoderma transiens*	2		Agaricomycetes	Polyporales	Hyphodermataceae	Hyphoderma
*Hyphodontia arguta*	15		Agaricomycetes	Hymenochaetales	Schizoporaceae	Hyphodontia
*Hyphodontia aspera*	1		Agaricomycetes	Hymenochaetales	Schizoporaceae	Hyphodontia
*Hyphodontia breviseta*	5		Agaricomycetes	Hymenochaetales	Schizoporaceae	Hyphodontia
*Hyphodontia crustosa*	76		Agaricomycetes	Hymenochaetales	Schizoporaceae	Hyphodontia
*Hyphodontia erastii*	1		Agaricomycetes	Hymenochaetales	Schizoporaceae	Hyphodontia
*Hyphodontia incrustata*	1		Agaricomycetes	Hymenochaetales	Schizoporaceae	Hyphodontia
*Hyphodontia microspora*	2		Agaricomycetes	Hymenochaetales	Schizoporaceae	Hyphodontia
*Hyphodontia nespori*	4		Agaricomycetes	Hymenochaetales	Schizoporaceae	Hyphodontia
*Hyphodontia pallidula*	12		Agaricomycetes	Hymenochaetales	Schizoporaceae	Hyphodontia
*Hyphodontia pruni*	2		Agaricomycetes	Hymenochaetales	Schizoporaceae	Hyphodontia
*Hyphodontia quercina*	2		Agaricomycetes	Hymenochaetales	Schizoporaceae	Hyphodontia
*Hyphodontia radula*	17		Agaricomycetes	Hymenochaetales	Schizoporaceae	Hyphodontia
*Hyphodontia sambuci*	33		Agaricomycetes	Hymenochaetales	Schizoporaceae	Hyphodontia
*Hyphodontia* sp.	7		Agaricomycetes	Hymenochaetales	Schizoporaceae	Hyphodontia
*Hyphodontia spathulata*	5		Agaricomycetes	Hymenochaetales	Schizoporaceae	Hyphodontia
*Hyphodontia subalutacea*	4		Agaricomycetes	Hymenochaetales	Schizoporaceae	Hyphodontia
*Hyphodontia tuberculata*	1		Agaricomycetes	Hymenochaetales	Schizoporaceae	Hyphodontia
*Hyphodontia verruculosa*	1		Agaricomycetes	Hymenochaetales	Schizoporaceae	Hyphodontia
*Hypochniciellum ovoideum*	1		Agaricomycetes	Amylocorticiales	Amylocorticiaceae	Hypochniciellum
*Hypochnicium geogenium*	3		Agaricomycetes	Polyporales	Podoscyphaceae	Hypochnicium
*Hypochnicium wakefieldiae*	10		Agaricomycetes	Polyporales	Podoscyphaceae	Hypochnicium
*Inonotus cuticularis*	1		Agaricomycetes	Hymenochaetales	Hymenochaetaceae	Inonotus
*Inonotus hispidus*	4		Agaricomycetes	Hymenochaetales	Hymenochaetaceae	Inonotus
*Inonotus lonicerinus*	2		Agaricomycetes	Hymenochaetales	Hymenochaetaceae	Inonotus
*Inonotus obliquus*	3	3	Agaricomycetes	Hymenochaetales	Hymenochaetaceae	Inonotus
*Inonotus rheades*	4	3	Agaricomycetes	Hymenochaetales	Hymenochaetaceae	Inonotus
*Irpex lacteus*	25	15	Agaricomycetes	Polyporales	Meruliaceae	Irpex
*Junghuhnia nitida*	5	1	Agaricomycetes	Polyporales	Steccherinaceae	Junghuhnia
*Lachnella alboviolascens*	1		Agaricomycetes	Agaricales	Tricholomataceae	Lachnella
*Lachnella* sp.	4		Agaricomycetes	Agaricales	Tricholomataceae	Lachnella
*Laetiporus sulphureus*	3	11	Agaricomycetes	Polyporales	Fomitopsidaceae	Laetiporus
*Lagarobasidium detriticum*	1		Agaricomycetes	Hymenochaetales	Schizoporaceae	Lagarobasidium
*Laxitextum bicolor*	2		Agaricomycetes	Russulales	Gloeocystidiellaceae	Laxitextum
*Lentaria patouillardii*	3		Agaricomycetes	Gomphales	Lentariaceae	Lentaria
*Lenzites betulina*	1		Agaricomycetes	Polyporales	Coriolaceae	Lenzites
*Lenzites warnieri*	7	4	Agaricomycetes	Polyporales	Coriolaceae	Lenzites
*Leptosporomyces galzinii*	1		Agaricomycetes	Atheliales	Atheliaceae	Leptosporomyces
*Leptosporomyces mundus*	1		Agaricomycetes	Atheliales	Atheliaceae	Leptosporomyces
*Leucogyrophana mollusca*	12		Agaricomycetes	Boletales	Boletales fam incertae sedis	Leucogyrophana
*Leucogyrophana pinastri*	1		Agaricomycetes	Boletales	Boletales fam incertae sedis	Leucogyrophana
*Loweomyces fractipes*	2		Agaricomycetes	Polyporales	Steccherinaceae	Loweomyces
*Macrotyphula fistulosa*	4	4	Agaricomycetes	Agaricales	Typhulaceae	Macrotyphula
*Macrotyphula juncea*	10		Agaricomycetes	Agaricales	Typhulaceae	Macrotyphula
*Maireina maxima*	2		Agaricomycetes	Agaricales	Niaceae	Maireina
*Mensularia radiata*	18	6	Agaricomycetes	Hymenochaetales	Hymenochaetaceae	Mensularia
*Merismodes fasciculata*	5		Agaricomycetes	Agaricales	Tricholomataceae	Merismodes
*Merulius tremellosus*	26	18	Agaricomycetes	Polyporales	Meruliaceae	Merulius
*Metulodontia nivea*	1		Agaricomycetes	Russulales	Peniophoraceae	Metulodontia
*Mucronella calva*	4		Agaricomycetes	Agaricales	Clavariaceae	Mucronella
*Mucronella flava*	1		Agaricomycetes	Agaricales	Clavariaceae	Mucronella
*Mycoacia columellifera*	1		Agaricomycetes	Polyporales	Meruliaceae	Mycoacia
*Mycoacia fuscoatra*	2		Agaricomycetes	Polyporales	Meruliaceae	Mycoacia
*Mycoacia uda*	7		Agaricomycetes	Polyporales	Meruliaceae	Mycoacia
*Mycoaciella bispora*	4		Agaricomycetes	Polyporales	Meruliaceae	Mycoaciella
*Odontia ferruginea*	1		Agaricomycetes	Thelephorales	Thelephoraceae	Odontia
*Odontia fibrosa*	1		Agaricomycetes	Thelephorales	Thelephoraceae	Odontia
*Oxyporus corticola*	21	3	Agaricomycetes	Hymenochaetales	Schizoporaceae	Oxyporus
*Oxyporus latemarginatus*	10		Agaricomycetes	Hymenochaetales	Schizoporaceae	Oxyporus
*Oxyporus obducens*	5	1	Agaricomycetes	Hymenochaetales	Schizoporaceae	Oxyporus
*Oxyporus similis*	1		Agaricomycetes	Hymenochaetales	Schizoporaceae	Oxyporus
*Oxyporus* sp.	1		Agaricomycetes	Hymenochaetales	Schizoporaceae	Oxyporus
*Paullicorticium pearsonii*	1		Agaricomycetes	Polyporales	Polyporales fam incertae sedis	Paullicorticium
*Peniophora cinerea*	34		Agaricomycetes	Russulales	Peniophoraceae	Peniophora
*Peniophora erikssonii*	5		Agaricomycetes	Russulales	Peniophoraceae	Peniophora
*Peniophora incarnata*	5		Agaricomycetes	Russulales	Peniophoraceae	Peniophora
*Peniophora laeta*	2		Agaricomycetes	Russulales	Peniophoraceae	Peniophora
*Peniophora lilacea*	20		Agaricomycetes	Russulales	Peniophoraceae	Peniophora
*Peniophora limitata*	16	4	Agaricomycetes	Russulales	Peniophoraceae	Peniophora
*Peniophora lycii*	10		Agaricomycetes	Russulales	Peniophoraceae	Peniophora
*Peniophora nuda*	30	1	Agaricomycetes	Russulales	Peniophoraceae	Peniophora
*Peniophora pini*	2		Agaricomycetes	Russulales	Peniophoraceae	Peniophora
*Peniophora polygonia*	3	2	Agaricomycetes	Russulales	Peniophoraceae	Peniophora
*Peniophora quercina*	19	25	Agaricomycetes	Russulales	Peniophoraceae	Peniophora
*Peniophora rufomarginata*	11	13	Agaricomycetes	Russulales	Peniophoraceae	Peniophora
*Peniophora violaceolivida*	4		Agaricomycetes	Russulales	Peniophoraceae	Peniophora
*Peniophorella pallida*	8		Agaricomycetes	Hymenochaetales	Hymenochaetales fam incertae sedis	Peniophorella
*Peniophorella praetermissa*	44		Agaricomycetes	Hymenochaetales	Hymenochaetales fam incertae sedis	Peniophorella
*Peniophorella pubera*	43		Agaricomycetes	Hymenochaetales	Hymenochaetales fam incertae sedis	Peniophorella
*Peniophorella tsugae*	1		Agaricomycetes	Hymenochaetales	Hymenochaetales fam incertae sedis	Peniophorella
*Perenniporia narymica*	1		Agaricomycetes	Polyporales	Ganodermataceae	Perenniporia
*Phaeolus schweinitzii*	2		Agaricomycetes	Polyporales	Fomitopsidaceae	Phaeolus
*Phanerochaete cumulodentata*	5		Agaricomycetes	Polyporales	Meruliaceae	Phanerochaete
*Phanerochaete deflectens*	1		Agaricomycetes	Polyporales	Meruliaceae	Phanerochaete
*Phanerochaete jose-ferreirae*	2		Agaricomycetes	Polyporales	Meruliaceae	Phanerochaete
*Phanerochaete livescens*	3		Agaricomycetes	Polyporales	Meruliaceae	Phanerochaete
*Phanerochaete sanguinea*	1		Agaricomycetes	Polyporales	Meruliaceae	Phanerochaete
*Phanerochaete sordida*	21		Agaricomycetes	Polyporales	Meruliaceae	Phanerochaete
*Phanerochaete* sp.	1		Agaricomycetes	Polyporales	Meruliaceae	Phanerochaete
*Phanerochaete tuberculata*	16		Agaricomycetes	Polyporales	Meruliaceae	Phanerochaete
*Phanerochaete velutina*	9		Agaricomycetes	Polyporales	Meruliaceae	Phanerochaete
*Phellinus igniarius*	8	14	Agaricomycetes	Hymenochaetales	Hymenochaetaceae	Phellinus
*Phellinus pomaceus*	4	2	Agaricomycetes	Hymenochaetales	Hymenochaetaceae	Phellinus
*Phellinus populicola*	2		Agaricomycetes	Hymenochaetales	Hymenochaetaceae	Phellinus
*Phellinus rhamni*	6		Agaricomycetes	Hymenochaetales	Hymenochaetaceae	Phellinus
*Phellinus tremulae*	5		Agaricomycetes	Hymenochaetales	Hymenochaetaceae	Phellinus
*Phlebia acerina*	7	1	Agaricomycetes	Polyporales	Meruliaceae	Phlebia
*Phlebia albida*	1		Agaricomycetes	Polyporales	Meruliaceae	Phlebia
*Phlebia bresadolae*	1		Agaricomycetes	Polyporales	Meruliaceae	Phlebia
*Phlebia lilascens*	2		Agaricomycetes	Polyporales	Meruliaceae	Phlebia
*Phlebia radiata*	9	5	Agaricomycetes	Polyporales	Meruliaceae	Phlebia
*Phlebia subochracea*	10		Agaricomycetes	Polyporales	Meruliaceae	Phlebia
*Phlebia tremelloidea*	1		Agaricomycetes	Polyporales	Meruliaceae	Phlebia
*Phlebia tuberculata*	1		Agaricomycetes	Polyporales	Meruliaceae	Phlebia
*Phlebiopsis gigantea*	3	4	Agaricomycetes	Polyporales	Meruliaceae	Phlebiopsis
*Phylloporia ribis*	1	4	Agaricomycetes	Hymenochaetales	Hymenochaetaceae	Phylloporia
*Piptoporus betulinus*	2	11	Agaricomycetes	Polyporales	Fomitopsidaceae	Piptoporus
*Piptoporus quercinus*	1		Agaricomycetes	Polyporales	Fomitopsidaceae	Piptoporus
*Polyporus alveolaris*	5	5	Agaricomycetes	Polyporales	Polyporaceae	Polyporus
*Polyporus arcularius*	6	5	Agaricomycetes	Polyporales	Polyporaceae	Polyporus
*Polyporus ciliatus*	1		Agaricomycetes	Polyporales	Polyporaceae	Polyporus
*Polyporus squamosus*		4	Agaricomycetes	Polyporales	Polyporaceae	Polyporus
*Polyporus varius*	2		Agaricomycetes	Polyporales	Polyporaceae	Polyporus
*Porodaedalea pini*	4	4	Agaricomycetes	Hymenochaetales	Hymenochaetaceae	Porodaedalea
*Porostereum spadiceum*	21	5	Agaricomycetes	Polyporales	Meruliaceae	Porostereum
*Postia alni*	15		Agaricomycetes	Polyporales	Fomitopsidaceae	Postia
*Postia floriformis*	1		Agaricomycetes	Polyporales	Fomitopsidaceae	Postia
*Postia leucomallella*	8		Agaricomycetes	Polyporales	Fomitopsidaceae	Postia
*Postia stiptica*	1		Agaricomycetes	Polyporales	Fomitopsidaceae	Postia
*Postia tephroleuca*	1		Agaricomycetes	Polyporales	Fomitopsidaceae	Postia
*Pseudoinonotus dryadeus*	2		Agaricomycetes	Hymenochaetales	Hymenochaetaceae	Pseudoinonotus
*Radulodon aneirinus*	5		Agaricomycetes	Polyporales	Cerrenaceae	Radulodon
*Radulomyces confluens*	107	2	Agaricomycetes	Agaricales	Pterulaceae	Radulomyces
*Radulomyces molaris*	21	6	Agaricomycetes	Agaricales	Pterulaceae	Radulomyces
*Ramaria abietina*	1		Agaricomycetes	Gomphales	Gomphaceae	Ramaria
*Ramaria corrugata*	1		Agaricomycetes	Gomphales	Gomphaceae	Ramaria
*Ramaria flaccida*	9		Agaricomycetes	Gomphales	Gomphaceae	Ramaria
*Ramaria ochracea*	1		Agaricomycetes	Gomphales	Gomphaceae	Ramaria
*Ramaria stricta*	1		Agaricomycetes	Gomphales	Gomphaceae	Ramaria
*Resupinatus poriaeformis*	4		Agaricomycetes	Agaricales	Tricholomataceae	Resupinatus
*Rigidoporus pouzarii*	2		Agaricomycetes	Polyporales	Meripilaceae	Rigidoporus
*Rigidoporus sanguinolentus*	5		Agaricomycetes	Polyporales	Meripilaceae	Rigidoporus
*Royoporus badius*	3		Agaricomycetes	Polyporales	Polyporaceae	Royoporus
*Sarcodontia pachyodon*	1		Agaricomycetes	Polyporales	Meruliaceae	Sarcodontia
*Schizophyllum amplum*	13	10	Agaricomycetes	Agaricales	Schizophyllaceae	Schizophyllum
*Schizophyllum commune*	17	46	Agaricomycetes	Agaricales	Schizophyllaceae	Schizophyllum
*Schizopora flavipora*	21	6	Agaricomycetes	Hymenochaetales	Schizoporaceae	Schizopora
*Schizopora paradoxa*	18	4	Agaricomycetes	Hymenochaetales	Schizoporaceae	Schizopora
*Scopuloides hydnoides*	10		Agaricomycetes	Polyporales	Meruliaceae	Scopuloides
*Scytinostroma hemidichophyticum*	6		Agaricomycetes	Russulales	Lachnocladiaceae	Scytinostroma
*Serpula himantioides*	5	1	Agaricomycetes	Boletales	Serpulaceae	Serpula
*Sistotrema binucleosporum*	1		Agaricomycetes	Cantharellales	Cantharellales fam incertae sedis	Sistotrema
*Sistotrema brinkmannii*	36		Agaricomycetes	Cantharellales	Cantharellales fam incertae sedis	Sistotrema
*Sistotrema diademiferum*	2		Agaricomycetes	Cantharellales	Cantharellales fam incertae sedis	Sistotrema
*Sistotrema oblongisporum*	1		Agaricomycetes	Cantharellales	Cantharellales fam incertae sedis	Sistotrema
*Sistotrema octosporum*	1		Agaricomycetes	Cantharellales	Cantharellales fam incertae sedis	Sistotrema
*Sistotrema porulosum*	1		Agaricomycetes	Cantharellales	Cantharellales fam incertae sedis	Sistotrema
*Sistotrema resinicystidium*	1		Agaricomycetes	Cantharellales	Cantharellales fam incertae sedis	Sistotrema
*Sistotrema sernanderi*	2		Agaricomycetes	Cantharellales	Cantharellales fam incertae sedis	Sistotrema
*Sistotrema* sp.	3		Agaricomycetes	Cantharellales	Cantharellales fam incertae sedis	Sistotrema
*Sistotremastrum niveocremeum*	6		Agaricomycetes	Trechisporales	Trechisporales fam incertae sedis	Sistotremastrum
*Sistotremastrum suecicum*	16		Agaricomycetes	Trechisporales	Trechisporales fam incertae sedis	Sistotremastrum
*Sistotremella hauerslevii*	1		Agaricomycetes	Trechisporales	Hydnodontaceae	Sistotremella
*Skeletocutis amorpha*	3		Agaricomycetes	Polyporales	Fomitopsidaceae	Skeletocutis
*Skeletocutis carneogrisea*	17	2	Agaricomycetes	Polyporales	Fomitopsidaceae	Skeletocutis
*Skeletocutis nivea*	6		Agaricomycetes	Polyporales	Fomitopsidaceae	Skeletocutis
*Steccherinum bourdotii*	2		Agaricomycetes	Polyporales	Steccherinaceae	Steccherinum
*Steccherinum fimbriatum*	21	6	Agaricomycetes	Polyporales	Steccherinaceae	Steccherinum
*Steccherinum ochraceum*	13	6	Agaricomycetes	Polyporales	Steccherinaceae	Steccherinum
*Steccherinum oreophilum*	1		Agaricomycetes	Polyporales	Steccherinaceae	Steccherinum
*Stereum gausapatum*	2	2	Agaricomycetes	Russulales	Stereaceae	Stereum
*Stereum hirsutum*	32	24	Agaricomycetes	Russulales	Stereaceae	Stereum
*Stereum sanguinolentum*	4	4	Agaricomycetes	Russulales	Stereaceae	Stereum
*Stereum subtomentosum*	33	20	Agaricomycetes	Russulales	Stereaceae	Stereum
*Stypella dubia*	1		Agaricomycetes	Auriculariales	Hyaloriaceae	Stypella
*Stypella grilletii*	1		Agaricomycetes	Auriculariales	Hyaloriaceae	Stypella
*Subulicystidium longisporum*	20		Agaricomycetes	Russulales	Peniophoraceae	Subulicystidium
*Thanatephorus fusisporus*	3		Agaricomycetes	Cantharellales	Ceratobasidiaceae	Thanatephorus
*Thelephora terrestris*	5	6	Agaricomycetes	Thelephorales	Thelephoraceae	Thelephora
*Thelephorales* sp.	2		Agaricomycetes	Thelephorales	Thelephorales fam incertae sedis	Thelephorales gen incertae sedis
*Tomentella badia*	1		Agaricomycetes	Thelephorales	Thelephoraceae	Tomentella
*Tomentella ferruginea*	2		Agaricomycetes	Thelephorales	Thelephoraceae	Tomentella
*Tomentella italica*	1		Agaricomycetes	Thelephorales	Thelephoraceae	Tomentella
*Tomentella pilosa*	2		Agaricomycetes	Thelephorales	Thelephoraceae	Tomentella
*Tomentella radiosa*	2		Agaricomycetes	Thelephorales	Thelephoraceae	Tomentella
*Tomentella* sp.	1		Agaricomycetes	Thelephorales	Thelephoraceae	Tomentella
*Tomentella spinosispora*	7		Agaricomycetes	Thelephorales	Thelephoraceae	Tomentella
*Tomentella stuposa*	3		Agaricomycetes	Thelephorales	Thelephoraceae	Tomentella
*Tomentella sublilacina*	4		Agaricomycetes	Thelephorales	Thelephoraceae	Tomentella
*Tomentella subtestacea*	1		Agaricomycetes	Thelephorales	Thelephoraceae	Tomentella
*Tomentellopsis bresadolana*	26		Agaricomycetes	Thelephorales	Thelephoraceae	Tomentellopsis
*Tomentellopsis echinospora*	1		Agaricomycetes	Thelephorales	Thelephoraceae	Tomentellopsis
*Tomentellopsis pulchella*	3		Agaricomycetes	Thelephorales	Thelephoraceae	Tomentellopsis
*Tomentellopsis* sp.	19		Agaricomycetes	Thelephorales	Thelephoraceae	Tomentellopsis
*Trametes hirsuta*	7	9	Agaricomycetes	Polyporales	Coriolaceae	Trametes
*Trametes ljubarskyi*	4	1	Agaricomycetes	Polyporales	Coriolaceae	Trametes
*Trametes ochracea*	22	19	Agaricomycetes	Polyporales	Coriolaceae	Trametes
*Trametes pubescens*	1		Agaricomycetes	Polyporales	Coriolaceae	Trametes
*Trametes suaveolens*	2		Agaricomycetes	Polyporales	Coriolaceae	Trametes
*Trametes trogii*	13	14	Agaricomycetes	Polyporales	Coriolaceae	Trametes
*Trametes versicolor*	9		Agaricomycetes	Polyporales	Coriolaceae	Trametes
*Trametopsis cervina*	3		Agaricomycetes	Polyporales	Meruliaceae	Trametopsis
*Trechinothus smardae*	1		Agaricomycetes	Agaricomycetes ord incertae sedis	Agaricomycetes fam incertae sedis	Trechinothus
*Trechispora alnicola*	3		Agaricomycetes	Trechisporales	Hydnodontaceae	Trechispora
*Trechispora cohaerens*	19		Agaricomycetes	Trechisporales	Hydnodontaceae	Trechispora
*Trechispora confinis*	2		Agaricomycetes	Trechisporales	Hydnodontaceae	Trechispora
*Trechispora farinacea*	4		Agaricomycetes	Trechisporales	Hydnodontaceae	Trechispora
*Trechispora hypoleucum*	1		Agaricomycetes	Trechisporales	Hydnodontaceae	Trechispora
*Trechispora kavinioides*	1		Agaricomycetes	Trechisporales	Hydnodontaceae	Trechispora
*Trechispora microspora*	2		Agaricomycetes	Trechisporales	Hydnodontaceae	Trechispora
*Trechispora nivea*	2		Agaricomycetes	Trechisporales	Hydnodontaceae	Trechispora
*Trechispora praefocata*	1		Agaricomycetes	Trechisporales	Hydnodontaceae	Trechispora
*Trechispora stevensonii*	15		Agaricomycetes	Trechisporales	Hydnodontaceae	Trechispora
*Trichaptum biforme*	6	2	Agaricomycetes	Hymenochaetales	Hymenochaetales fam incertae sedis	Trichaptum
*Trichaptum fuscoviolaceum*	29	20	Agaricomycetes	Hymenochaetales	Hymenochaetales fam incertae sedis	Trichaptum
*Tubulicrinis calothrix*	1		Agaricomycetes	Hymenochaetales	Tubulicrinaceae	Tubulicrinis
*Tubulicrinis strangulatus*	1		Agaricomycetes	Hymenochaetales	Tubulicrinaceae	Tubulicrinis
*Tubulicrinis subulatus*	1		Agaricomycetes	Hymenochaetales	Tubulicrinaceae	Tubulicrinis
*Tulasnella albida*	7		Agaricomycetes	Cantharellales	Tulasnellaceae	Tulasnella
*Tulasnella brinkmannii*	1		Agaricomycetes	Cantharellales	Tulasnellaceae	Tulasnella
*Tulasnella deliquescens*	1		Agaricomycetes	Cantharellales	Tulasnellaceae	Tulasnella
*Tulasnella eichleriana*	11		Agaricomycetes	Cantharellales	Tulasnellaceae	Tulasnella
*Tulasnella hyalina*	1		Agaricomycetes	Cantharellales	Tulasnellaceae	Tulasnella
*Tulasnella pallida*	12		Agaricomycetes	Cantharellales	Tulasnellaceae	Tulasnella
*Tulasnella pinicola*	1		Agaricomycetes	Cantharellales	Tulasnellaceae	Tulasnella
*Tulasnella pruinosa*	1		Agaricomycetes	Cantharellales	Tulasnellaceae	Tulasnella
*Tulasnella saveloides*	6		Agaricomycetes	Cantharellales	Tulasnellaceae	Tulasnella
*Tulasnella* sp.	5		Agaricomycetes	Cantharellales	Tulasnellaceae	Tulasnella
*Tulasnella thelephorea*	5		Agaricomycetes	Cantharellales	Tulasnellaceae	Tulasnella
*Tulasnella tomaculum*	1		Agaricomycetes	Cantharellales	Tulasnellaceae	Tulasnella
*Tulasnella violea*	8		Agaricomycetes	Cantharellales	Tulasnellaceae	Tulasnella
*Typhula erythropus*	4		Agaricomycetes	Agaricales	Typhulaceae	Typhula
*Typhula euphorbiae*	1		Agaricomycetes	Agaricales	Typhulaceae	Typhula
*Typhula micans*	1		Agaricomycetes	Agaricales	Typhulaceae	Typhula
*Typhula setipes*	25		Agaricomycetes	Agaricales	Typhulaceae	Typhula
*Typhula* sp.	10		Agaricomycetes	Agaricales	Typhulaceae	Typhula
*Typhula sphaeroidea*	5		Agaricomycetes	Agaricales	Typhulaceae	Typhula
*Tyromyces chioneus*	3		Agaricomycetes	Polyporales	Fomitopsidaceae	Tyromyces
*Vararia ochroleuca*	2		Agaricomycetes	Russulales	Lachnocladiaceae	Vararia
*Vuilleminia comedens*	24	18	Agaricomycetes	Corticiales	Vuilleminiaceae	Vuilleminia
*Vuilleminia coryli*	5	5	Agaricomycetes	Corticiales	Vuilleminiaceae	Vuilleminia
*Vuilleminia cystidiata*	2	1	Agaricomycetes	Corticiales	Vuilleminiaceae	Vuilleminia
*Vuilleminia pseudocystidiata*	1		Agaricomycetes	Corticiales	Vuilleminiaceae	Vuilleminia
*Xenasmatella* sp.	1		Agaricomycetes	Agaricomycetes ord incertae sedis	Xenasmataceae	Xenasmatella
*Xenasmatella vaga*	5		Agaricomycetes	Agaricomycetes ord incertae sedis	Xenasmataceae	Xenasmatella

**Table 4.** Re-identifications of the eastern Ukrainian fungal collections based on recent taxonomical revisions. For all such species, the specimens were studied and cited personally by the authors of the new names (but see comment for *Antrodia hyalina*).

**Original identification**	**Current name**	**Reference**
*Antrodia pulvinascens*	*Antrodia hyalina*	V. Spirin, personal communication; see also Spirin et al. 2013
*Eichleriella deglubens*	*Heteroradulum kmetii*	Malysheva and Spirin 2017
*Phanerochaete magnoliae*	*Phanerochaete cumulodentata*	Volobuev et al. 2015
*Phanerochaete sordida*	*Phanerochaete livescens*	Volobuev et al. 2015
*Rigidoporus crocatus*	*Rigidoporus pouzarii*	Vampola and Vlasák 2012

## Footnotes

*1 open for registered users of PlutoF platform (Abarenkov et al. 2010, https://plutof.ut.ee)
